# King Edward's Hospital Fund for London

**Published:** 1911-04-01

**Authors:** 


### KING EDWARD'S HOSPITAL FUND FOR LONDON.

The annual meeting of the Governors and General Council of King Edward's Hospital Fund for London, to receive the accounts and the report of the General Council for the year 1910, was held at St. James's Palace on Tuesday morning, March 28, His Serene Highness the Duke of Teck being in the chair.

Those present were: Viscount Iveagh and the Speaker of the House of Commons (the Right Hon. James W. Lowther, M.P.), the Bishop of London, the Rev. T. Bowman Stephenson, D.D., the Chief Rabbi (the Rev. Dr. Adler), the Earl of Bessborough, Lord Rothschild, Lord Revelstoke, the President of the Hospital Sunday and Hospital Saturday Funds (the Right Hon. the Lord Mayor), the President of the Royal College of Surgeons (Mr. Henry T. Butlin), the Right Hon. C. Stuart-Wortley, K.C., M.P., the Right Hon. Sir Ernest Cassel, the Right Hon. Sir Joseph Dimsdale, Bart., the Right Hon. Sir Savile Crossley, Bart. (hon. secretary), Lieut.-Colonel the Right Hon. Sir Arthur Bigge, Sir W. S. Church, Bart., Sir Henry Burdett, Sir Horace Marshall, Sir W. J. Collins, Sir Cooper Perry, Sir John Craggs, Mr. J. Danvers Power, Rev. Canon Barnett, Dr. Edwin Freshfield, Mr. Frederick M. Fry (hon. secretary), Mr. William Latham, K.C., Mr. Albert G. Sandeman.

Lord Rothschild (the honorary treasurer), in presenting the account of receipts and expenditure and the balance-sheet for the year ending December 31, 1910, said it would be noticed that they had distributed last year no less a sum than £155,000. He ought to state that they became possessed of the reversion of an estate, and one of the annuitants enjoying the income during her lifetime died after a long illness, the expenses of which could hardly have been met without assistance from the Fund. The Council were advised that the Fund could not be drawn upon for this, but one subscriber was good enough to authorise a portion of his subscription to be devoted to the purpose, and that was done. In connection with the balance-sheet it should be stated that the income derived from non-trust funds was £17,000 in excess of what it would have been if the money had been invested in trust funds. In dealing with these large sums the total expenditure was only £3,000, or 1½ per cent. upon the revenue.

The Duke of Teck formally moved the adoption of the accounts.

The resolution having been seconded by the Lord Mayor (Sir T. Vezey Strong), was then put and carried unanimously.

The Council's Report.

Sir Savile Crossley (honorary secretary) presented the draft report of the Council for the year 1910 as follows:—

The Late King.

For the second time since the foundation of King Edward's Hospital Fund the General Council have to mourn the loss of a beloved Sovereign and Patron.

The following resolution passed by the Executive Committee on May 12, 1910, expresses the profound sorrow of all connected with the Fund at the death of His late Majesty King Edward VII., its illustrious founder:—

"The Executive Committee of King Edward's Hospital Fund for London on behalf of the General Council desire to record their profound sorrow at the great loss which all those associated with the Fund have sustained by the death of their beloved Sovereign King Edward VII.

"It was on his initiative that the Commemoration of the sixtieth year of the reign of Queen Victoria took the form of a scheme for the assistance of the hospitals of London, and the Fund which he thus founded and which bears his name is therefore a lasting monument to his constant sympathy with the poor and suffering.

"To his active personal share in its administration during the years in which he was President, the Fund owed its rapid establishment in public confidence, and he gave repeated assurances of the interest with which he watched its continued progress under his illustrious successor.

"The Committee respectfully desire to tender their deepest sympathy to His Majesty King George V. and His Gracious Consort in the sad bereavement sustained by their Majesties, and to offer the humble assurance of their loyalty and devotion."

The Presidency of the Fund.

As a result of the death of His late Majesty the Fund has also been deprived of the incalculable advantage of the personal supervision and direction of its affairs exercised for nine years by His present Majesty when Prince of Wales and President of the Fund. The Council are glad to report that His Majesty has graciously been pleased to become Patron of the Fund, and to express his unabated interest in all that concerns its welfare.

In June last His Majesty intimated his intention to increase his annual subscription to the Fund from £500 to £1,000. Her Majesty the Queen and His Royal Highness the Prince of Wales have also been graciously pleased to increase their subscriptions.

During the interval which under present circumstances must elapse before another Royal President can be appointed, the duties of that office are, in accordance with the provisions of the King Edward's Hospital Fund for London Act, exercised by three Governors appointed by His Majesty on the recommendation of the Lord Chancellor, the Prime Minister, and the Governor of the Bank of England. Pursuant to this provision, His Majesty has appointed as Governors His

Serene Highness the Duke of Teck, Viscount Iveagh, and the Speaker of the House of Commons.

### Receipts.

The total receipts for the year 1910 were £205,169 16s. 2d. This sum was made up as follows: Donations, £17,767 12s. 2d.; contributions to capital, £32,596 12s. 3d., including a legacy of £32,153 19s., together with £147 10s. net profit on realisation of securities; annual subscriptions, £26,728 16s. 9d.; contribution of the League of Mercy, £18,000; from the Lewis Estate, £10,000; legacies (other than legacies to capital), £22,941 9s. 8d.; interest from investments, £76,935 5s. 4d.; together with £200 from the Trustees of the Bawden Fund.

### Grants.

The amount distributed was £155,000, being an increase of £5,000 over the sum distributed in 1909. Of this the hospitals received £150,000, while the remaining £5,000 was distributed amongst consumption sanatoria and convalescent homes taking London patients. The total sum distributed amongst hospitals, convalescent homes, and consumption sanatoria since the foundation of the Fund (after deducting grants to the extent of £5,659 16s. 7d. that have been allowed to lapse) is £1,288,916 8s. 5d.

### Administration Expenses.

Since the inauguration of the Fund the amount spent on administration has been £1 4s. 11d. per cent. of the total amount received.

### Legacies, Donations, and Subscriptions.

Amongst the legacies received during the year were payments of £32,153 19s. on account of the residue of the estate of the late Mrs. Hames, placed to capital in accordance with the wishes of the testator, £10,000 from the late Miss Elizabeth Chambers, £4,919 10s. 6d. from the late Mr. Richard Clarke, a further £3,200 on account of the legacy of the late Miss E. S. Wolfe, and £2,000 from the late Miss Ellen Morrison. The Council desires to acknowledge specially the receipt of an annual subscription of £5,000 from Mr. Walter Morrison, and an anonymous donation of £10,000. The amounts received in donations and subscriptions, excluding gifts to capital, show, as compared with 1909, an increase of £7,735 19s. 5d.

### League of Mercy.

The League of Mercy has this year contributed £18,000. During the eleven years of its existence the League has furnished in all £153,000 to the Fund. The Council desire to record their grateful appreciation of the labour of the voluntary workers who have contributed towards the collection of this large sum.

### Work of the Fund's Visitors.

Every eligible hospital and consumption sanatorium which applied for a grant was inspected and reported upon by the Fund's Visitors, consisting as heretofore of one medical and one layman in each case.

### Beds at Consumption Sanatoria.

The proposal by the Convalescent Homes Committee to secure beds at consumption sanatoria which could be placed at the disposal of hospitals in London for the accommodation of suitable cases urgently requiring country treatment has now taken practical shape. Grants have been made on this basis by means of which eighteen beds have been secured at three sanatoria. It is hoped that the scheme may be extended in future, and the Council have authorised the Convalescent Homes Committee, if necessary, to consider applications for grants on suitable terms towards the capital cost of providing beds for the purpose, where this can only be done by means of additions to the existing accommodation.

### Co-operative Purchase by Hospitals.

In January 1910 the question of the possible advantages of co-operative purchase by hospitals again came under the consideration of the Executive Committee, largely as the result of the establishment of a scheme of this kind in New York. A Sub-committee, consisting of Mr. John G. Griffiths, F.C.A., Chairman, the Hon. Sydney Holland, and the Hon. Arthur Stanley, was appointed to consider the question. The report of this Sub-committee, which suggests the possibility of co-operation by groups of hospitals in different parts of London, has been circulated to the hospitals with an offer of assistance from the Fund, if desired, in connection with any experiments in this direction.

The same Sub-committee also considered the question of forms of tender, and appended to their report a number of specimen forms.

### Statistics of Expenditure.

The annual Statistical Report prepared by the Fund, which began with sixteen hospitals only, has been again enlarged. The report published in 1910 dealt with the expenditure of ninety-nine hospitals, while similar particulars in respect of another group of eight institutions were circulated privately. With the addition of this group to the published report, the series will deal with nearly every hospital in London which adopts the Revised Uniform System of Accounts, and the assistance which the issue of comparative figures renders towards the achievement of economy will be proportionately increased.

### Out-Patient Inquiry.

The appointment of the Special Committee to inquire into the method prevailing in the London voluntary hospitals with regard to the admission of out-patients (foreshadowed by His present Majesty when Prince of Wales and President of the Fund) was definitely announced by the Governors at the meeting of the Council in December. The Council consider that the Fund is fortunate in having obtained the assistance of three such eminent Commissioners as Lord Mersey, the Chairman of the Committee, Lord Northcote, and the Bishop of Stepney, in its efforts to secure a solution of the questions at issue.

### Hospital Extension or Reconstruction.

The Distribution Committee have continued to make a careful examination of all schemes for the extension or reconstruction of hospitals, submitted in accordance with the request of the Council. The Fund thus uses its best endeavours to ensure that when capital expenditure of this kind is undertaken, due attention is paid to economy, efficiency, the prospect of funds for future maintenance, and the relation of each scheme to the general question of hospital accommodation in London.

### The Council's Loss.

The Council desire to place on record their sense of the loss the Fund has sustained by the death of Mr. Hugh C. Smith, who had served the Fund for many years in the various capacities of Trustee, Member of Council, Chairman of Executive Committee, and latterly, Member of Finance Committee. The Council also deeply regret the loss of one of their first members by the death of Sir John Aird.

### Auditors.

The President of the Local Government Board, in pursuance of his powers under the Act, has appointed Messrs. Deloitte, Plender, Griffiths and Co. to audit the accounts of the Fund for the year.

### Thanks to the Press.

The Council are obliged to the Press for the services they have rendered in the past, and continue to rely upon their kindly co-operation in obtaining for the Fund wider publicity and support.

St. James's Palace, March 28, 1911.

His Serene Highness the Duke of Teck, in moving the adoption of the report, said:

My Lords and Gentlemen,—It was the custom of His present Majesty, when Prince of Wales and President of the Fund, to review the events of the year, not at this annual meeting, but at the distribution meeting in the previous December. I propose to follow this practice, and as at the last meeting I mentioned all the principal points dealt with in the annual report which has just been read, I will now only make a brief allusion to one subject—the Special Committee on the out-patient question. That Committee, under the able chairmanship of Lord Mersey, has been sitting regularly since January, and has made considerable progress with the receipt of evidence. I am sure we are much indebted, not only to the three Commissioners who are devoting so much time and trouble to this important work, but also to the numerous witnesses, both hospital representatives and others interested in the question, who are giving the Committee the benefit of their special knowledge and experience. I now beg to move the adoption of the report.

The Earl of Bessborough seconded the motion, which was carried unanimously.

Sir William Church moved the adoption of the following report of the Distribution Committee upon the question of the suspended grant to St. George's Hospital.

Distribution Committee's Report upon the Question of the Suspended Grant to St. George's Hospital.

At the meeting of the Governors and General Council on December 14, 1910, the Report of the Distribution Committee was adopted, recommending that a grant of £2,000 should be made to St. George's Hospital, but that the grant should be retained until the Fund is satisfied that the relations between Hospital and Medical School are in accordance with the principles intimated by the Fund in May, 1909. Paragraph (10) of that Report as amended and adopted by the Council and published is as follows:—

Extract from Report of Distribution Committee, December, 1910.

(10) In the case of St. George's Hospital, the Committee have had under their special consideration the appearance, in the general accounts of the Hospital for 1909, of certain payments to the School which were stated to be for work done for the Hospital and which were formerly paid out of a discretionary fund. The enquiries made by the Committee as to the legitimacy of this change confirmed the statement in the Annual Report of the Hospital that the payments in question were made on account of definite work done, chiefly pathological and bacteriological examinations, in connection with the actual treatment of patients. That is, the payments were for the kind of work which is described in Appendix III. of the Report of Sir Edward Fry's Committee as work which would have to be paid for in any case, and which would have to be taken into account in assessing the payments and receipts on either side.* Such work, moreover, differs entirely from the general benefits, described in paragraphs 13 to 22 of the Report as being conferred by Medical Schools on Hospitals, and as being fairly set off against the benefits conferred by the Hospitals on the Schools. The opinion of the Fund, that, on the principles laid down by Sir Edward Fry's Committee, specific payments for such definite work done in respect of the treatment of patients, whether made to the School or to any other agency, could be charged to the general funds of the Hospital, while no other payments to the Schools could be so charged, was notified to St. George's, in reply to a question from the Hospital, by the Executive Committee in May 1909. But on looking into the method by which the amount fairly chargeable to the Hospital on account of this work had been arrived at, the Distribution Committee came to the conclusion that the apportionment of the expenses of the laboratories as between the treatment of patients and medical education required investigation, and they have invited the Hospital to submit further details in order that the Committee may satisfy themselves that the payment made to the School bears a proper proportion to such definite work done for the Hospital.

In the meantime they recommend that the grant to the Hospital this year should be retained until the Fund is satisfied that the financial relations between Hospital and School are in accordance with the principles intimated by the Fund in May 1909, and that the general account of the Hospital in respect of 1909, as well as 1910, is clear from any payment on account of medical education.

The Distribution Committee have been in communication with the Authorities of the Hospital, and a Sub-committee of their members have visited the Hospital and inspected the Clinical Laboratories, Museum, and School Buildings, and they now desire to lay before the Governors and General Council the following report:—

The payments by the Hospital in 1909 in respect of the specific work in question consist of the following items:—Salaries, £1,075; Cash payment, £550; Rent remitted, £250, Total, £1,875. The Committee decided at the outset to make enquiries as to the cost of similar work at other Hospitals, for it is evident that if the cost to the Hospital of the laboratories is largely in excess proportionately of the cost at other comparable Hospitals, the difference requires investigation.

The Committee therefore endeavoured to obtain information enabling them to compare the cost of work done in the interests of the patients in the Clinical Laboratories at St. George's with that done at similar institutions. In reply to their enquiries they received confidential returns from nineteen institutions in London and the provinces, including eleven London Hospital with Medical Schools.

The returns, however, in reference to six Institutions were in such a form as to be of no use for the purpose of the investigation. From the figures supplied by the remaining Institutions and St. George's Hospital it appeared that the cost of laboratory investigations, etc., at St. George's Hospital worked out at about two and a half times the average, if St. George's was included, or slightly over two and three quarter times, if St. George's was excluded.

Differences of cost in this connection may be due either to differences in the relative amount of the total expenditure on the laboratory work or to differences in the method of its apportionment as between Hospital and Medical School. At St. George's the sum paid by the Hospital appears from the returns submitted to represent about four-fifths of the total cost, while the School pays about one-fifth.

Before discussing these figures it appears necessary to place before the Council certain changes which have taken place at St. George's Hospital and its Medical School since Sir Edward Fry's Committee reported.

Up to the year 1906 the laboratories attached to the Hospital were used by the School for the purposes of teaching the earlier subjects of professional study (Chemistry, Physics, Biology, Anatomy, Physiology, and Pharmacology) as well as for any purely hospital work necessary in the interests of the patients.

In 1906 the Medical School decided to abolish the teaching of the preliminary and intermediate subjects of the medical curriculum at the Hospital, and entered into an arrangement with the London University that these subjects should be taught to their students in the laboratories of the University of London.

The changes which have taken place in the medical and surgical treatment of patients during recent years necessitate a much larger amount of laboratory investigation than was formerly the case, and the Governing Body of St. George's Hospital decided to retain the Laboratories for purely clinical purposes, in which the work necessary for the treatment of the patients in or attending the Hospital could be carried out, and in which the students at their school could be instructed in the most modern methods of carrying out such investigations.

As a result of an interview with a deputation from the Hospital, the Committee informed St. George's on November 16, 1910, that in their view the method of apportioning the expenses of these laboratories, upon which the figures then submitted were based, was faulty, and that the remission of rent to the School should not have been left out of the calculation. The Committee further considered that the payment was excessive, that the apportionment between the Hospital and the Medical School should be revised, and that no payments over and above specific payment for definite work done in respect of the treatment of patients, calculated on the revised method, should be made out of the general fund. On November 30, the Hospital submitted a fresh arrangement of the figures based upon a different method of apportionment and including a payment for rent.

The Distribution Committee have not undertaken a minute examination of this revised basis of apportionment, nor do they desire to call in question the *bona-fides* of those responsible for it, though the position may be maintained that the larger part of the expenses of the Pathological Museum should not be borne by the Hospital, that a reasonable rent should be charged for the use by Students of the Hospital laboratories and the School buildings, and, finally, that the department of clinical investigation may have been planned and staffed upon its present basis, not only for the benefit of patients, but as an attraction to Students to join the Hospital.

Instead of entering upon these highly contentious topics, the Distribution Committee prefer to base their report upon the broad ground of comparative cost, and, as there is no reason to suppose that the work done at St. George's differs markedly in quality or quantity from that done at other London Hospitals of equal repute, they have come to the conclusion that the Fund ought to mark its disapproval of an expenditure so far in excess of the average, whether such excess arises from extravagance, from a contribution to the Medical School, or from the Clinical Laboratories being designed not only for Hospital requirements, but also for purposes of research.

In making this recommendation, however, the Committee desire to state that in their opinion the certificate given to the Fund by the Treasurer of St. George's Hospital, to the effect that no payments have been made to or on account of the Medical School for the purposes of medical education, was given in absolute good faith. In a matter of such complexity, the Committee are not surprised to find that different

* The following is an extract from this Appendix, which is referred to by Sir Edward Fry's Committee (Report, par. 9), as "The Third Appendix, which we adopt as part of our Report":—

"It must be remembered that, apart from these [i.e. the general services rendered by the Schools to the Hospitals], which are separately dealt with in the Report (par. 21), the schools, in most cases, undertake many specific laboratory examinations solely for the purpose of helping the physicians and surgeons to form correct diagnoses in the exclusive interests of the patients, and that these would have to be paid for by the hospitals in any case. Where such examinations are made in a laboratory entirely maintained by a school, it is obvious that, in any exact assessment of the payments and receipts on either side, they would have to be taken into account."

conclusions as to the proper apportionment of this expenditure should have been reached by different persons, whose *bona fides* is equally unquestioned. Their own conclusion, arrived at after a very careful consideration of all the aspects of the problem, is that a larger fraction of the expenditure on the Clinical Laboratories and the School buildings should have been borne by the School, and that the School should repay to the Hospital in respect of the year 1909 (and if necessary 1910), the difference between the sum of £1,000 and the charge borne by the Hospital in respect of specific work done for the Hospital patients, which amounted in 1909 to £1,875.

Sir Ernest Cassel seconded the adoption of the report.

In reply to a question by Sir William Collins, Sir William Church pointed out that the adoption of this report did not involve any departure from the rule passed by the Council in accordance with the recommendation of Sir Edward Fry's Committee, viz., that no grant should be made to any hospital which made payments to the account of its medical school out of general funds subscribed for the relief of the sick poor.

The motion having been supported by Sir Henry Burdett, was put and carried unanimously.

The Bishop of London, in moving a vote of thanks to the Duke of Teck for presiding, said there was no one in whom the Council had more confidence, and no one who took greater interest in hospital work.

The resolution was seconded by Mr. C. Stuart-Wortley and carried unanimously.

His Serene Highness the Duke of Teck having briefly acknowledged the vote of thanks, the proceedings then terminated.

